# Normal D-Dimer Plasma Level in a Case of Acute Thrombosis Involving Intramuscular Gastrocnemius Vein

**DOI:** 10.7759/cureus.20153

**Published:** 2021-12-04

**Authors:** Hany A Zaki, Amr Elmoheen, Abdallah M Elsafti Elsaeidy, Ahmed E Shaban, Eman E Shaban

**Affiliations:** 1 Emergency Medicine, Hamad Medical Corporation, Doha, QAT; 2 Emergency Medicine, Mansoura University Faculty of Medicine, Mansoura, EGY; 3 Internal Medicine, Mansoura General Hospital, Mansoura, EGY; 4 Internal Medicine, Mansoura University Faculty of Medicine, Mansoura, EGY; 5 Cardiology, Aljufairi Diagnostic and Therapeutic Hospital, Doha, QAT

**Keywords:** clinical probability rule, intramuscular gastrocnemius vein, pulmonary embolism (pe), deep venous thrombosis (dvt), venous thromboembolism (vte), d-dimer

## Abstract

Venous thromboembolism (VTE) is a major cause of morbidity and mortality among hospitalized patients. Studies have reported an incidence of deep venous thrombosis to be as high as 50%, especially after craniotomy. Several factors are involved in the alteration of the specificity and sensitivity of D-dimer testing. These include symptom duration, the extent of fibrinolytic and thrombosis activity, anticoagulant therapy, comorbidity associated with medical or surgical illness, cancer, inflammatory diseases, old age, postpartum, and pregnancy period, as well as previous VTE. Several studies have shown the high sensitivity of the D-dimer test (>95%) in pulmonary embolism or acute deep venous thrombosis. The cut-off value is usually within the 500 µg FEU/L range, ruling out acute VTE, especially in patients with low or intermediate clinical probability. Patients who present with a high D-dimer level may necessitate an intense diagnostic approach, the pretest probability notwithstanding. Herein, we present a case of a 52-year-old male patient who presented with a normal D-dimer level in deep venous thrombosis.

## Introduction

D-dimer plays an essential role as an initial screening test for the diagnosis of venous thromboembolism (VTE) patients in the emergency department. The incidence of VTE increases progressively with age [1]. The cumulative event rate exceeds 10% by 80 years of age [2] and is associated with substantial mortality and morbidity in the absence of treatment [3]. Clinical symptoms are usually not sufficiently specific for the establishment or exclusion of diagnosis [4]. Objective testing is required to verify clinical suspicion. These aid the management decisions, although every imaging technique currently in use has practical or clinical limitations.

D-dimer may be detected in patients suffering deep venous thrombosis (DVT) because it serves as a marker of endogenous fibrinolysis [5]. This test, known for its high negative predictive value (NPV), is carried out to provide a cost-effective and fast way to triage patients who have the thromboembolic phenomenon. Although the NPV of D-dimer may be on the high side, patients having a positive D-dimer have to undergo further imaging to determine whether they have the VTE or not. This article discusses the case of an elderly DVT patient with an average D-dimer level, including a review of D-dimer levels, factors that influence testing of D-dimer, and its role in the evaluation of recurrent DVT patients.

## Case presentation

A 52-year-old male patient known to have type II diabetes mellitus and essential hypertension was presented to the emergency unit of our facility with severe pain in the right foot and ankle and tenderness due to non-traumatic cause, which started early in the morning after prayers. The pain increased with movement and eased when the patient rested. No other limb involvement was recorded. There was also no previous episode of the same complaint. There was a conspicuous absence of chest pain or shortness of breath (SOB), calf muscle pain, or injuries. There were also no respiratory symptoms or fever, recent hospital admission, significant allergy, or previous hospital admission. There was also no recent travel or history of immobilization. DVT was previously non-existent in their family.

The patient's vital signs upon admission to the emergency department were as follows: temperature oral was 37.1°C, heart rate apical was 92 beats per minute (bpm), respiratory rate was 18 breaths per minute, systolic blood pressure (SBP) was 182/100 mmHg, and SpO_2_ was 98%. Physical examination revealed that the patient appears apyrexial, no (jaundice, anemia, cyanosis, clubbing, or lymphadenopathy), no skin rash, neck or back rigidity. Central nervous system review showed the patient was alert, Glasgow Coma Scale (GCS) was 15/15, no focal neurology, and no facial palsy; motor power five over five in all limbs, no motor or sensory deficit; no abnormal gait, no cerebellar signs, normal coordination. Cardiovascular system review showed that the pulse was regular with a typical character, jugular venous pressure (JVP) not raised. The first and second heart sounds were normal with no murmurs. Mild non-pitting right ankle edema was present. Respiratory system examination revealed no increased work of breathing, normal bilateral chest expansion, and percussion notes resonant. Chest sounds equal and bilateral, no added sounds. SpO_2_ 98% on room air. Abdominal examination revealed that the abdomen is not distended, soft, lax, and no tenderness, no guarding, or rigidity, and hernial orifices were normal. The extremities showed tenderness and edema on the right foot and ankle, reduced range of movement because of the pain with intact neurovascular, warm, good peripheral circulation, sensation, and motor power. No redness or hotness was found.

**Figure 1:**
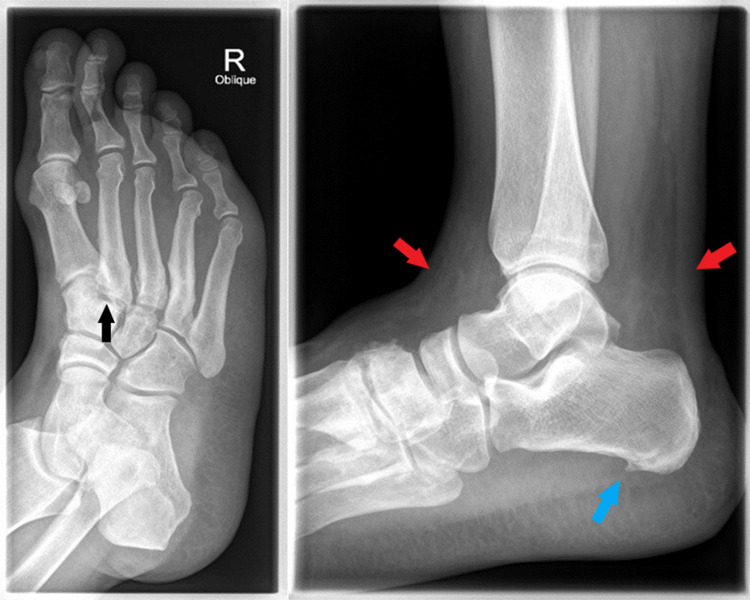
Right foot (at the left side) and right ankle (at the right side) showed no obvious fracture. Degenerative changes were seen involving the tarsometatarsal joint (black arrow) and bony spurs at the calcaneus (blue arrow), with significant soft tissue swelling involving the right ankle (red arrows).

The emergency department planned x-rays on the right foot and ankle, blood investigations, emergency management (analgesia), and reassessment. X-rays of the foot and ankle showed no apparent bony injuries or fractures. The x-rays showed degenerative changes involving tarsometatarsal joints and bony spurs at the calcaneus, with significant soft tissue swelling involving the right ankle (Figure 1).

**Figure 2:**
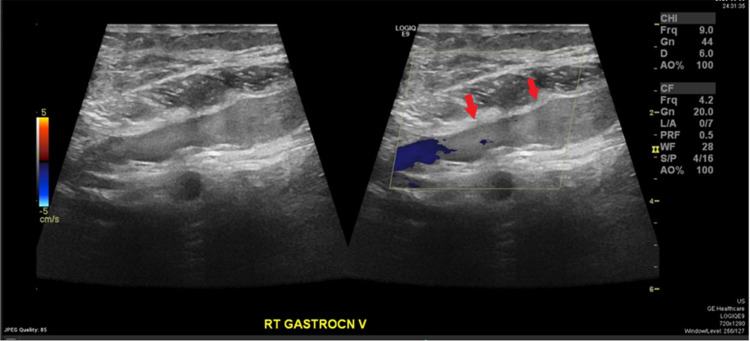
Doppler ultrasound image showed distended non-compressible gastrocnemius vein without appreciating flow (red arrows).

**Figure 3:**
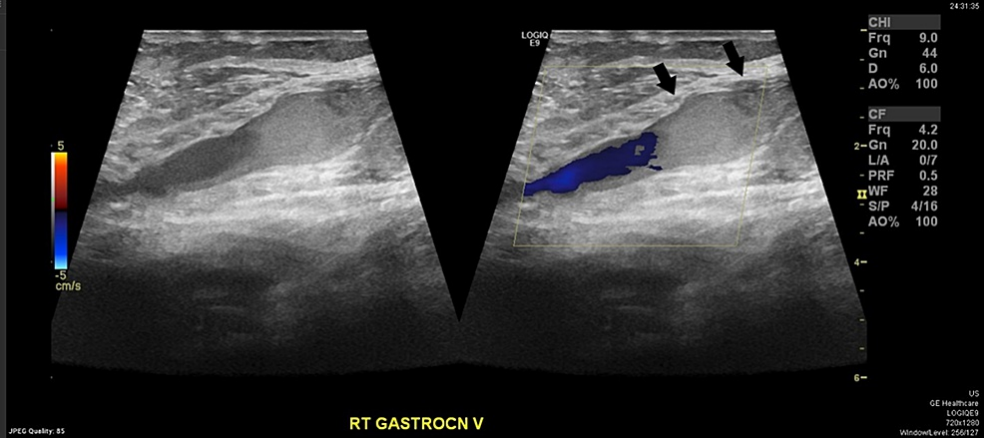
Doppler ultrasound image showed distended non-compressible gastrocnemius vein without appreciating the flow (black arrows).

**Figure 4:**
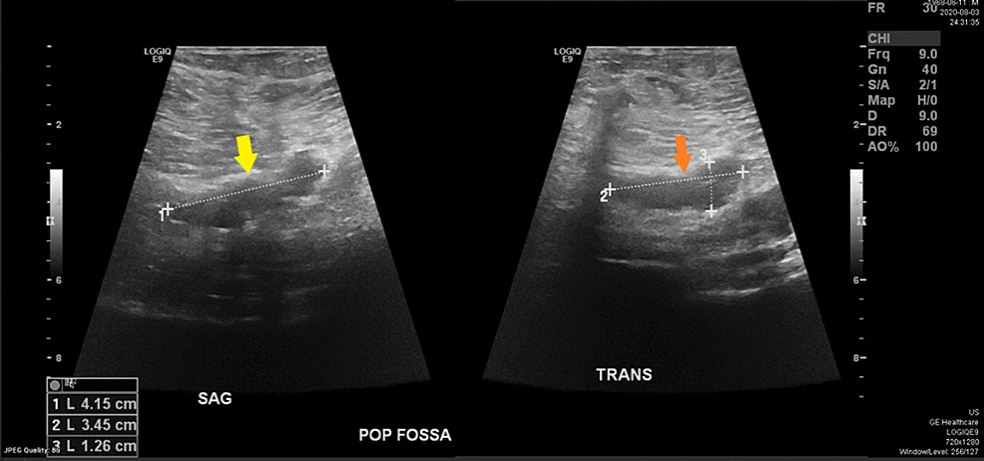
Doppler ultrasound image of the popliteal fossa in both sagittal (yellow arrow) and transverse view (orange arrow) showed backer cyst of 4.15x3.45x1.26 cm.

Blood investigations were all unremarkable including the normal serum D-dimer level 320 mg/L (D-dimer normal rage: <500 ng/mL). Clinical judgment suspects deep venous thrombosis as one of the differential diagnoses regarding the presenting case, so Doppler ultrasound on the same affected limb (right) was done and showed the following results: intramuscular calf muscle (gastrocnemius) vein distended and non-compressible with no flow appreciated suggesting acute thrombosis (Figures 2, 3). Also, a baker cyst of 4.15x3.45x1.26 cm was seen (Figure 4).

**Figure 5:**
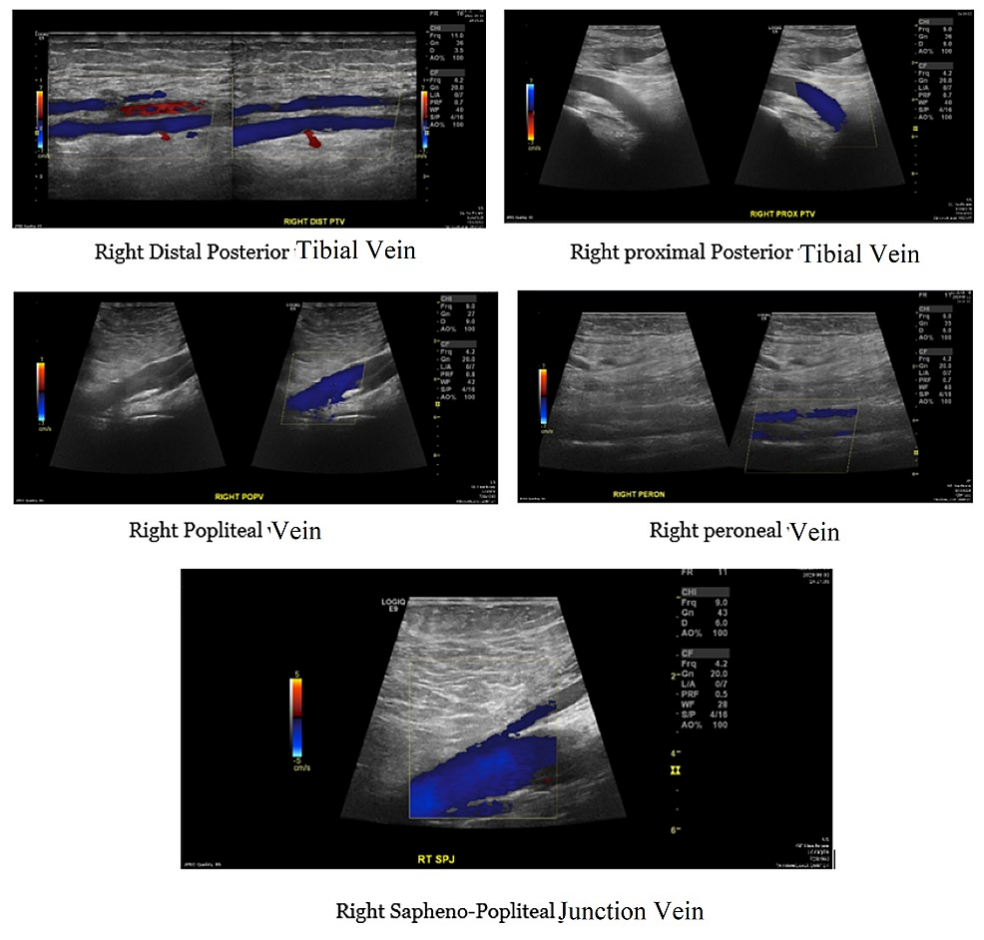
The right distal and proximal posterior tibial, popliteal, peroneal, and saphenopopliteal junction showed normal color flow.

The right external iliac vein shows normal color flow. The right common femoral, greater saphenous, superficial femoral, and popliteal veins show normal color flow and compressibility. The visualized right peroneal and posterior tibial veins show normal color flow (Figure 5).

The patient was admitted under the internal medicine specialty and started on enoxaparin according to the patient’s weight (134 kg according to the last measurement) plus warfarin 10 mg for three to six months. After three days from hospital admission, the patient was discharged and suggested to follow-up at the medicine outpatient department (OPD) of the warfarin clinic for thrombophilia workup.

## Discussion

Clinicians must understand that the D-dimer level goes up in many situations. Physiologic factors that contribute to increasing D-dimer level include puerperium and pregnancy, old age (>65 years), cigarette smoking, African American heritage, recent trauma, and the postoperative period [6]. Pathologic causes for increased D-dimer levels include thrombosis, cardiovascular disease, renal disease, liver disease, infections, malignancy, and chronic inflammatory diseases. Several strategies have been outlined for VTE diagnoses such as testing for D-dimer after negative imaging to identify patients requiring a new specific evaluation, combined D-dimer testing with clinical probability, and a test for the exclusion of VTE in patients with a positive test [6].

It is important to note that in the emergency unit, a D-dimer levels test is done to rule out a VTE diagnosis in low-risk patients. A 2004 meta-analysis by Stein et al. including over 31 studies to determine D-dimer accuracy testing in patients with VTE found that it had a 20-78% prevalence [7]. The average prevalence was 36%. In the study by Stein et al. patients with low clinical probability (LCP) or intermediate clinical probability (ICP) had a probability for false-positive D-dimer levels that ranged from 40% to 60%. It is worth mentioning that a negative D-dimer test effectively excludes VTE disease in a way that compares to duplex ultrasonography or a lung scan in LCP patients. A 2002 meta-analysis by Brown et al. assessing the use of D-dimer for VTE exclusion found that the prevalence was 17-58% [8]. In both aforementioned analyses, D-dimer testing sensitivity was 96% (95% confidence interval {CI}, 0.9-1.0) and 94% (95% CI, 0.8-0.9), respectively [7,8].

D-dimer has an extremely high sensitivity (>95%), excluding the cut-off value of acute VTE at 500 µg/L, especially in LCP or ICP patients [9]. A study carried out by Taira et al. found that LCP patients whose D-dimer levels were less than 500 ng DDU/mL may not require any additional or expensive imaging studies [10]. In a prospective study involving 270 patients with VTE, the ICP, LCP, and high clinical probability (HCP) were 64%, 11%, and 25%, respectively. For patients with suspected DVT, the HCP, ICP, and LCP were 59%, 32%, and 9%, respectively [11].

It is important to note that venous thromboembolism cannot be diagnosed solely on clinical presentation due to low sensitivity and lack of symptoms. Pulmonary embolism is a common and potentially mortal disorder, resulting in >250,000 hospitalizations yearly in the United States [12]. DVT and pulmonary embolism (PE) have a 20% prevalence [13]. If a VTE diagnosis is missed, it may result in sudden death, chronic dysfunction of the cardiopulmonary system, and impaired quality of life [14]. For those with suspected VTE, there is a need to identify those without the disease to obviate the need for anticoagulation therapy and risk of bleeding [15]. Additionally, excessive testing of patients for PE can be expensive and expose patients to the side effects of radiation, a false-positive diagnosis, intravenous contrast, and anticoagulation treatment. VTE risk factors include cancer, increasing age, acute infection, paralysis, long air travel, prolonged immobility, congestive heart failure, pregnancy or puerperium, varicose veins, hormonal treatment, dehydration, inflammatory bowel disease, previous VTE, rheumatologic disease, persistent D-dimer elevation, nephrotic syndrome, and atherosclerotic disease [16].

## Conclusions

Adequate efforts should be made to facilitate the implementation of VTE diagnostic strategies. The primary limitation of D-dimer testing is its application in unique clinical settings that lead to low specificities, as in elderly patients, oncology patients, and pregnant women. The high sensitivity of the test is, however, retained in these situations.
